# Ginsenoside Rg1 Acts as a Selective Glucocorticoid Receptor Agonist with Anti-Inflammatory Action without Affecting Tissue Regeneration in Zebrafish Larvae

**DOI:** 10.3390/cells9051107

**Published:** 2020-04-29

**Authors:** Min He, Mahmoud Halima, Yufei Xie, Marcel J. M. Schaaf, Annemarie H. Meijer, Mei Wang

**Affiliations:** 1 Institute of Biology Leiden, Leiden University, 2333CC Leiden, The Netherlands; heminccucm@hotmail.com (M.H.); m.halima@biology.leidenuniv.nl (M.H.); y.xie@biology.leidenuniv.nl (Y.X.); 2 Leiden University—European Center for Chinese Medicine, Leiden University, 2333CC Leiden, The Netherlands

**Keywords:** ginsenoside, Rg1, zebrafish, tail fin amputation, inflammation, glucocorticoid, leukocyte migration, tissue regeneration, selective glucocorticoid receptor agonist

## Abstract

Glucocorticoids are effective anti-inflammatory drugs, but their clinical use is complicated due to the wide range of side effects they induce. Patients requiring glucocorticoid therapy would benefit from more selective glucocorticoid receptor (GR) agonists, capable of attenuating the immune response without causing these side effects. Ginsenosides, such as the compound Rg1, are natural plant compounds with structural similarity to classical glucocorticoids and well-documented anti-inflammatory effects. Here, we have investigated the activity of the ginsenoside Rg1 using a zebrafish larval model, in which amputation of the tail fin allows us to assess drug effects on inflammation, while the ability to regenerate the wounded tissue serves as a readout for side effects. We found that Rg1 attenuates neutrophilic inflammation at the amputation site, similarly to a classical glucocorticoid, beclomethasone. Mutation of the Gr abolishes this anti-inflammatory effect of Rg1. Rg1 and beclomethasone differentially modulate gene expression, suggesting that Rg1 induces transrepression, but not transactivation, activity of Gr. Interestingly, we found no effect of Rg1 on tissue regeneration, whereas beclomethasone inhibits tissue regeneration entirely. We conclude that Rg1 is a promising candidate for development as a selective glucocorticoid drug, and that zebrafish larvae provide a useful model system for screening of such GR agonists.

## 1. Introduction

Inflammation is a critical biological response of the body required to deal with tissue damage and the re-establishment of homeostasis. Complex signaling pathways are involved in the inflammatory response, involving the activation of pattern recognition receptors, such as Toll-like receptors (TLRs) and leucine-rich-repeat-containing receptors (NLRs) [1,2], the activation of central immune regulators, such as Nuclear Factor-kappa B (NF-κB) and Activator Protein-1 (AP-1) [3], increased expression of proinflammatory cytokines, such as Tumor Necrosis Factor-alpha (TNF-α), Interleukin-1beta (IL-1β), Interleukin-6 (IL-6), and Interferon-gamma (IFN-γ), and the enhanced production of chemokines such as CXCL8 (IL-8), CCl2 and, CXCL11. As a result, immune cells such as neutrophils, macrophages, and lymphocytes migrate into the affected tissue to eliminate microbial invaders [4,5,6,7]. Neutrophils are the initial responders recruited to the injured tissue and mediate the proinflammatory response [8,9,10]. The recruitment of neutrophils further enhances the activation and migration of macrophages, which mediate the clearance of apoptotic neutrophils and boost the antimicrobial activity of neighboring immune cells [11,12]. Once inflammation is resolved, macrophages shift from an activated (M1) to a wound-healing (M2) phenotype and restore homeostasis [11,12]. However, in chronic inflammatory disorders, there is a failure to resolve inflammation, which can lead to tissue damage and loss of function. Long-term inflammation is associated with severe disorders, such as asthma, rheumatoid arthritis, insulin-dependent diabetes mellitus, colitis ulcerosa, and Crohn’s disease [13,14].

Presently, glucocorticoids (GCs) such as prednisolone, dexamethasone, hydrocortisone, and beclomethasone are frequently used for suppressing the overactive immune system in chronic inflammatory diseases, as these drugs can alter monocyte recirculation, induce lymphocyte death, and inhibit the production of proinflammatory cytokines and other effector molecules [15,16]. The effects of GCs are mediated by the glucocorticoid receptor (GR), which acts as a ligand-activated transcription factor modulating the transcription of many genes [17]. It regulates gene transcription through two main mechanisms: transactivation by binding to glucocorticoid response elements in the DNA, and transrepression by interaction with other transcription factors such as NF-κB and AP-1, thereby modulating their activity [18]. The transrepression activity is generally considered to be the basis of the anti-inflammatory actions of the activated GR [19]. Since the GR is expressed in a wide variety of cell types, GCs influence many different processes in our body, and have a wide range of side effects such as hyperglycemia, osteoporosis, hypertension, weight gain, muscle weakness, psychiatric disturbances, and diminished wound healing and tissue regeneration [14,20]. Therefore, there is an urgent need for the development of other classes of anti-inflammatory drugs, or more selective GR agonists that maintain the anti-inflammatory activity but lack the adverse effects on other body functions.

Ginseng Radix, which refers to the root of the plant *Panax ginseng* C.A. Mayer, also known as Chinese/Korean ginseng, is one of the most widely used herbal products in the world. Ginseng has been suggested to promote longevity and has a long history of applications in traditional Chinese medicine related to a range of biological activities, such as wound healing ability, anti-inflammatory activity, anticancer activity, vasorelaxation, and antioxidative effects [21]. Several studies have provided evidence that Ginseng Radix treatment modulates the immune response [21,22]. Ginsenosides are considered as the most important bioactive components in *P. ginseng*, with ginsenoside Rg1 being among the most abundant. Rg1 has been recognized as a steroid-like saponin, similar in structure to GCs, and it has been demonstrated that Rg1 is a functional ligand of the GR [23,24,25]. Different in vitro studies have demonstrated that Rg1 modulates the immune response [22,23,24], and it has been shown to inhibit acute and chronic inflammation in mice without causing hyperglycemia or osteoporosis [26].

Over the last two decades, the zebrafish has become widely utilized as a model organism in biomedical research, owing to the suitability of the embryonic and larval stages for genetic and chemical screening and for microscopic imaging of cellular behavior during development and disease [27]. The zebrafish has a single *gr* gene encoding a Gr structurally and functionally highly similar to the human GR [28,29,30]. The tail fin amputation model in larval zebrafish is a well-established system for anti-inflammatory drug screening, and it has been used to investigate the anti-inflammatory effect of GCs [17,31,32,33,34,35,36]. In this assay, the tail fins of three-day post fertilization (dpf) larvae were amputated. Subsequently, neutrophils and macrophages, the major leukocytes present at this stage of development [37], migrate towards the wounded area, and the number of migrating leukocytes is considered a measure for the severity of the inflammatory response. Within several days after the amputation, the tail fin will fully regenerate. GCs have been shown to inhibit the migration of neutrophils and decrease the amputation-induced increase in the expression of proinflammatory genes. Interestingly, they also inhibit the regeneration of the tail fin [17,32,33,34,35]. Therefore, the tail fin amputation model provides a convenient system to compare the anti-inflammatory potential of ginsenosides and classical GCs, while using the regenerative capacity of the wounded tissue as readout for the evaluation of side effects.

In the present study, we have investigated the anti-inflammatory properties of Rg1 using the zebrafish tail fin amputation model. Our results demonstrate that Rg1 has anti-inflammatory activity in this assay and that Gr activation mediates the anti-inflammatory effects of this compound, similarly to a classical GC, beclomethasone. Rg1 induced different alterations in the transcription of various immune-related genes and did not inhibit regeneration of the amputated tail fin of zebrafish, in contrast to beclomethasone. These data demonstrate that Rg1 has selective activity, which distinguishes this compound from classical GCs. We therefore suggest that Rg1 may be further developed as a selective GR agonist drug with effective anti-inflammatory action and reduced side effects.

## 2. Materials and Methods

### 2.1. Zebrafish Lines and Maintenance

Zebrafish (*Danio rerio*) were maintained and handled according to the guidelines from the Zebrafish Model Organism Database (http://zfin.org) and in compliance with the directives of the local animal welfare committee of Leiden University. They were exposed to a 14 h light and 10 h dark cycle to maintain circadian rhythmicity. Fertilization was performed by natural spawning at the beginning of the light period. Eggs were collected and raised at 28 °C in egg water (60 μg/mL Instant Ocean sea salts and 0.0025% methylene blue). Besides wild type zebrafish (AB/TL), the following zebrafish lines were used in this study: the double transgenic line *Tg*(*mpx:GFP^i114^/mpeg1:mCherry-F^umsF001^*) and the *gr^s357^* mutant line [38,39,40]. For generation of homozygous *gr^s357^* (referred to as *gr^−/−^*) and wild type (*gr^+/+^*) fish, heterozygous fish were in-crossed, and the offspring was genotyped. Subsequently, the *gr^−/−^* and *gr^+/+^* fish of this generation were raised separately and in-crossed to obtain the *gr^−/−^* and *gr^+/+^* larvae used in this study.

### 2.2. Fish Embryo Acute Toxicity Test (FET) for Rg1

A Fish Embryo Acute Toxicity Test (FET) was performed for Rg1. For this purpose, the guidelines of the Organization for Economic Cooperation and Development (OECD, No.236 [41]) were adapted according to a previously published protocol for testing of valuable compounds, such as nanoparticles [42], because of the relatively high costs for Rg1. A range of five test concentrations of Rg1 was used (100, 120, 150, 180, and 210 μM). Stock solutions were made in DMSO, and final dilutions in egg water (60 μg/mL Instant Ocean sea salts and 0.0025% methylene blue), such that final DMSO concentration were 0.01%. The following controls were used: a negative control (nC, egg water), a solvent control (sC, 0.01% DMSO in egg water), a positive control (pC, 4 mg/L 3,4-dichloroaniline in egg water), and 25 μM beclomethasone (which was used in other experiments as a control as well).

Embryos were collected around 1.5 hpf and distributed over standard 24 well plates (twenty embryos per well), with each well containing 2 mL of test solution. The transgenic line *Tg*(*mpx:GFP^i114^/mpeg1:mCherry-F^umsF001^*) was used for proper comparison with other experiments in this study. Five 24-well plates were prepared, each with five wells containing the Rg1 solutions (100, 120, 150, 180, and 210 μM), three wells for beclomethasone, two for nC and sC, and one for pC (on each plate, 11 wells remained empty). All solutions were refreshed daily.

The plates were kept at a temperature between 26 and 27 °C with dissolved oxygen above 70%, pH between 7.11 and 7.89, and exposed to a 12 h light and 12 h dark cycle. At 72 and 96 hpf, the average survival rate (in %) for all experimental groups was determined. Additionally, hatching was recorded at 48 and 72 hpf. Survival rates for the Rg1- and pC-treated groups from the five individual plates were averaged. The rates for the beclomethasone-, nC- and sC-treated wells (three, three, and two, respectively) were first averaged per plate, and subsequently the survival rates from the individual plates were averaged. Statistical analysis was performed using ANOVA, with Tukey’s post hoc comparisons. Hatching rates at 96 hpf were within the criteria for test validity (≥80% for nC and sC), as well as the survival rates (≥90% for nC and sC, ≤70% for pC). Finally, the LC50 for Rg1 was determined in the R software environment for statistical computing and graphics, using the ‘drc’ package [43]. Briefly, the survival rates were plotted against the Rg1 concentration and a curve was fitted, described by the following formula:

$$f\left(x\right)=\frac{100}{1+{\left(x/LC50\right)}^{b}}$$

where coefficient *b* denotes the steepness of the dose–response curve.

### 2.3. Tail Fin Amputation and Drug Treatments

Three-day-old larvae were utilized for the tail fin amputation experiments. In each experiment, three different treatment groups (20 larvae, unless otherwise indicated) were used: a vehicle (DMSO) treatment group (negative control), a group treated with beclomethasone (25 μM, positive control) [32], and a group treated with Rg1 (120 μM). All groups were pretreated with vehicle/beclomethasone/Rg1 for 2 h before tail fin amputations, and received the same treatment for 4 h after the amputation. Next, larvae were anesthetized in egg water containing 0.02% buffered amino benzoic acid ethyl ester (tricaine; Sigma-Aldrich, St. Louis, MO, USA). Larvae were placed on petri dishes coated with 2% agarose under a Leica M165C stereomicroscope, and the tail fins were partly amputated using a 1 mm sapphire blade (World Precision Instruments). For quantification of leukocyte migration, larvae were fixed overnight in 4% paraformaldehyde (PFA) at 4 °C. For gene expression analysis by qPCR, an additional nonamputated, vehicle-treated group was used, and all samples contained 15 larvae, which were collected in TRIzol reagent (Invitrogen, Waltham, MA, USA) and stored at −20 °C. For all experiments, three independent replicates were performed.

### 2.4. Visualization and Quantification of Macrophages and Neutrophils

**Figure 1:**
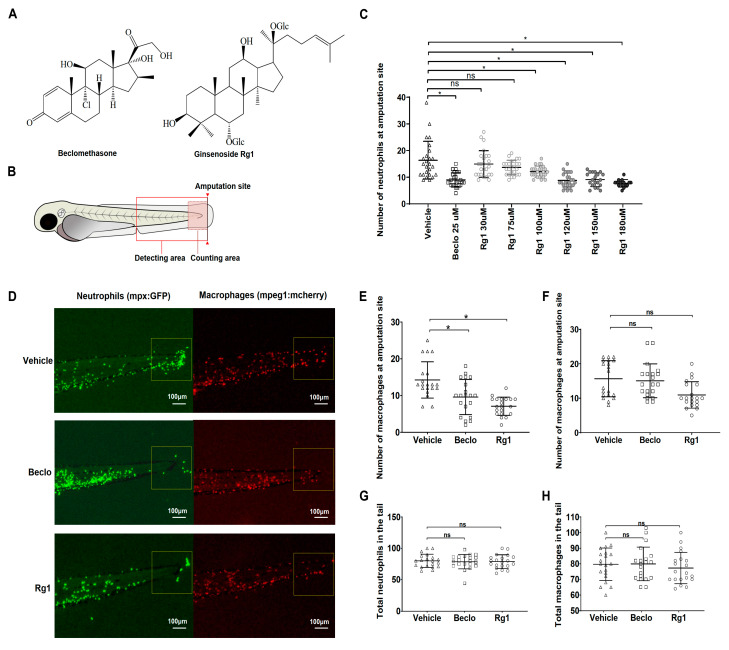
Inhibitory effects of Rg1 and beclomethasone on wounding-induced leukocyte migration. (**A**) The chemical structures of the classical GC beclomethasone, and the ginsenoside Rg1, which has a steroid-like skeleton consisting of four trans-rings, (three six-member cyclohexane rings and one five-member cyclopentane ring). (**B**) Schematic drawing of a zebrafish larva at 3 dpf, indicating the site of tail fin amputation, the region of microscopic imaging, and the area for quantification of neutrophils and macrophages (i.e., the counting area: posterior to the tail vein). (**C**) Dose-dependent anti-inflammatory effect of Rg1. Numbers of neutrophils that have migrated towards the amputated tail fin at 4 hpa and 2 h pre- and 4 h post-treatment with Rg1 or beclomethasone at the indicated concentrations. The graph shows data from an experiment with 25 larvae per group, representative of three experimental repeats. No mortality was observed at Rg1 concentrations up to 120 μM, while 20% and 40% of embryos on average died in the 150 μM and 180 μM groups. (**D**) Representative fluorescence microscopy images of wound-induced migration of neutrophils (in green fluorescence channel) and macrophages (in red fluorescence channel), in combination with vehicle, beclomethasone (Beclo), and Rg1 treatments. Scale bar: 100 μm. (**E**,**F**) Numbers of neutrophils (**E**) and macrophages (**F**) that have migrated towards the amputation site, after treatment of 3 dpf larvae with vehicle, Beclo, or Rg1. The graphs show data from an experiment with 20 larvae per group (each indicated as individual data points), representative of three experimental repeats. (**G**,**H**) The total number of neutrophils (**G**) and macrophages (**H**) present in the entire tail region (posterior to the yolk sac extension). ns—not significant. * *p* < 0.05.

Imaging of the *Tg*(*mpx:GFP^i114^/mpeg1:mCherry-F^umsF001^*) larvae was performed utilizing a LeicaMZ16FA fluorescence stereomicroscope supported by LAS 3.7 software. The macrophages were detected based on the red fluorescence of their mCherry label, and neutrophils were detected based on their green fluorescent GFP label. To quantify the number of macrophages and neutrophils recruited to the wounded area, the cells in a defined area of the tail (Figure 1B) were counted manually.

For the *gr^s35^* larvae, an additional TSA^®^ fluorescein detection kit (PerkinElmer, Waltham, MA, USA) was utilized in order to specifically stain neutrophils to enable visualization of these cells by fluorescence microscopy. Fixed larvae were washed 3 × 5 min in PBS containing 0.8% Triton X-100 (PBS-TX). Then, larvae were washed briefly in 100 μL of the Amp diluent of TSA kit. After that, 100 μL of a 1:50 dilution of TSA:Amp diluent was added. Next, larvae were incubated for 10 min at 28 °C in the dark. Then, larvae were washed 3 × 10 min in PBS-TX. Subsequently, samples were fixed 20 min in 4% PFA, and washed with PBS containing 0.1% Tween20.

### 2.5. Regeneration of Amputated Larvae Tail Fin

For the regeneration experiments, three groups of 2 dpf larvae, each group consisting of 30 larvae, were used for the tail fin amputation, and regeneration was determined at 5 dpf. The 2 dpf stage was chosen to reduce the number of animal experiments in the sense of EU Directive 2010/63/EU. Regeneration of the tail fin lasts three days, and by starting at 2 dpf, zebrafish could be studied until 120 dpf in this experiment. Until that stage they are not considered independently feeding, and therefore not subject to regulations for animal experimentation [44]. At 2 dpf, beclomethasone has a similar inhibitory effect on neutrophil migration after tail fin amputation as it does at 3 dpf (data not shown). Larvae from the *Tg*(*mpx:GFP^i114^/mpeg1:mCherry-F^umsF001^*) line were used in these experiments, although the fluorescence was not used as a readout. All groups were pretreated 2 h prior to the tail fin amputations as mentioned previously. After that, amputated larvae were treated for 4 h with either Rg1 or beclomethasone. After the 4 h treatment, fresh egg water was replaced again so that larvae stay and live with the normal environment for another three days before imaging. Three independent experiments were performed. A LeicaMZ16FA fluorescence stereomicroscope supported by the LAS version 3.7 software was utilized to determine the regeneration process of the amputated tail fins.

### 2.6. Quantitative PCR (qPCR) Analysis

For qPCR analysis, larvae were collected (15 per sample) in TRIzol lysis reagent (Invitrogen) for RNA isolation, which was performed using the miRNeasy mini kit (Qiagen, Venlo, The Netherlands), according to the manufacturer’s instructions. For cDNA synthesis, the iScript cDNA synthesis kit (Bio-Rad Laboratories, Hercules, CA, USA) was employed, using 1 µg of each RNA sample. Subsequently, 17 genes were analyzed using qPCR using the MyiQ single-color real-time PCR detection system (BioRad Laboratories). The analysis was performed in a total volume of 25 µL containing 5 µL cDNA, 5.5 µL water, 1 µL forward and 1 µL reverse primer (10µM), and 12.5 µL of 2× iQ SYBR Green Supermix (Bio-Rad Laboratories). PCR settings were: 95 °C for 3 min, 40 cycles of 15 s at 95.5 °C, 15 s at 60 °C, and 30 s at 72 °C. Cycle threshold (Ct) values were defined for each run. For every sample, The Ct value was deducted from the Ct value of a control sample. The fold alteration of gene expression was calculated and normalized to the expression levels of peptidylprolyl isomerase Ab (*ppial*) as a reference gene. The lid temperature was 105 °C. An additional protocol was included to determine the dissociation of the PCR products from 65 °C to 95 °C, permitting the recognition of the amplified products. Reactions were performed in duplicate. The sequences of all qPCR primers are presented in Table S1.

### 2.7. Statistical Analysis

**Figure 2:**
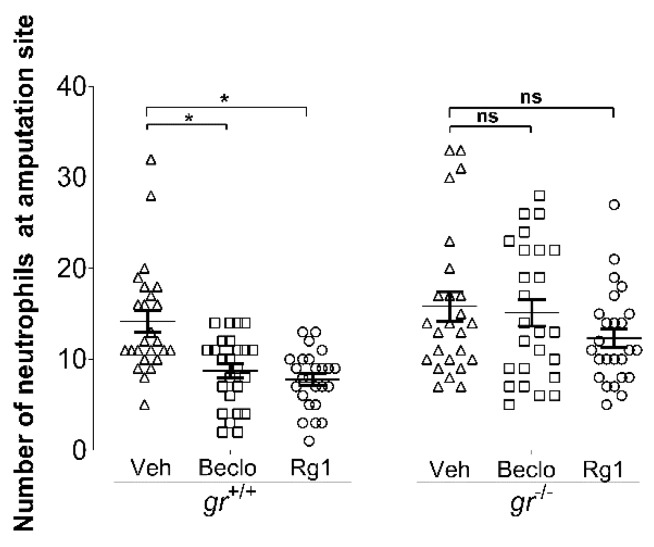
Glucocorticoid receptor (GR)-dependency of the anti-inflammatory effects of Rg1 and beclomethasone. Quantification of neutrophil migration towards the amputated site in the tail fin at 3 dpf in Gr mutant (*gr^−/−^*) and wild type (*gr^+/+^*) larvae, after treatment with vehicle, beclomethasone (Beclo), or Rg1. The graphs show data from an experiment with 25 larvae per group (each indicated as individual data points), representative of three experimental repeats. ns—not significant. * *p* < 0.05.

**Figure 3:**
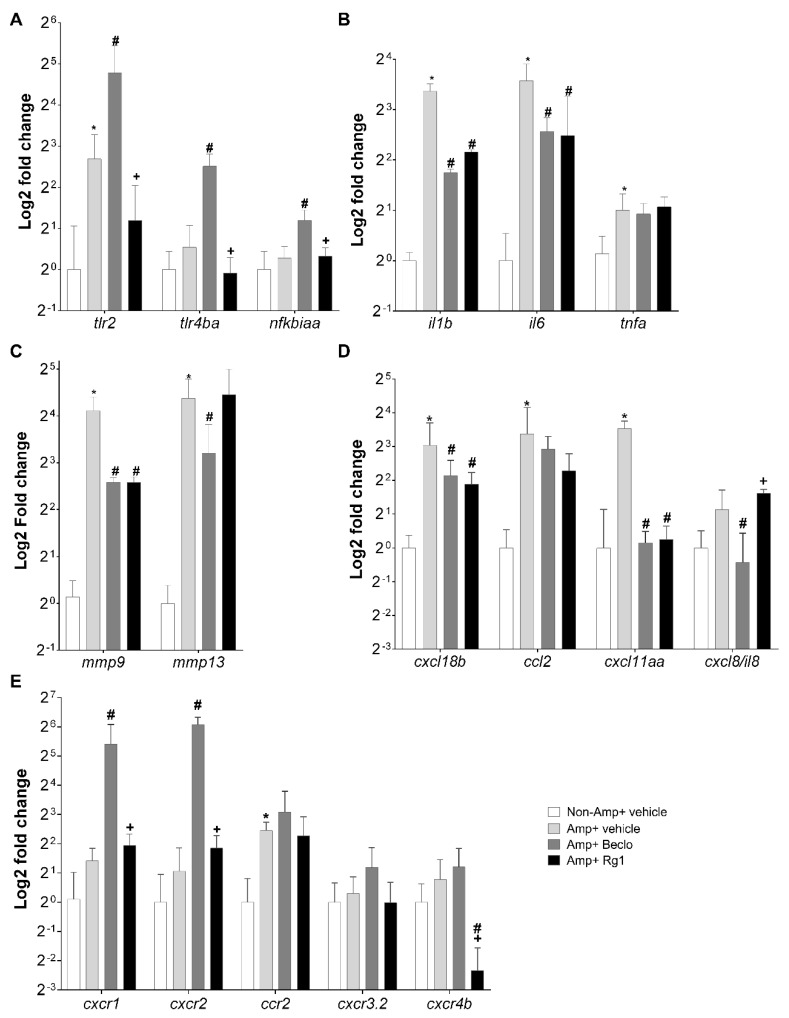
Differential effects of Rg1 and beclomethasone on inflammatory gene expression. Expression analysis by qPCR using total RNA from 3 dpf larvae with (Amp) or without (Non-Amp) amputation and treated with vehicle, beclomethasone (Beclo), or Rg1. (**A**) Genes encoding proteins in general inflammatory pathways (Toll-like receptors and NF-κB inhibitor α-like protein (**A**). (**B**) Genes encoding proinflammatory cytokines (Interleukins and Tnfα). (**C**) Genes encoding Matrix metalloproteinases (Mmps), implicated in inflammation and tissue regeneration. (**D**,**E**) Genes encoding neutrophil- or macrophage-related chemokines (**D**) or chemokine receptors (**E**). The relative expression values were normalized to those of *ppial* and are shown on a log2 scale. Bars represent the mean ± SEM of three independent experiments (each with technical duplicates). * *p* < 0.05 compared with the nonamputated vehicle group. ^#^ *p* < 0.01 compared with the amputated vehicle group. + *p* < 0.05 compared with the amputated Beclo group.

**Figure 4:**
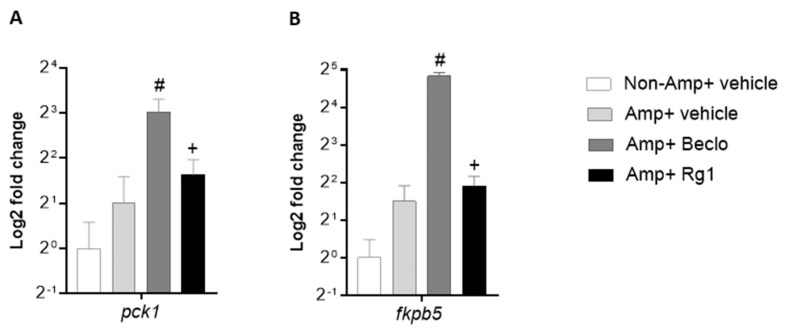
Differential effects of Rg1 and beclomethasone on the expression of endogenous Gr target genes. Expression analysis by qPCR using total RNA from 3 dpf larvae with (Amp) or without (Non-Amp) amputation and treated with vehicle, beclomethasone (Beclo), or Rg1. (**A**) Gene encoding Phosphoenolpyruvate Carboxykinase (Pck1). (**B**) Gene encoding FK506 binding protein 5 (Fkbp5). The relative expression values were normalized to those of *ppial* and are shown on a log2 scale. Bars represent the mean ± SEM of three independent experiments (each with technical duplicates). ^#^ *p* < 0.01 compared with the amputated vehicle group. + *p* < 0.05 compared with the amputated Beclo group.

Statistical analysis was performed using GraphPad Prism 7 by one-way (Figure 1D–G and Figure 2) and two-way (Figure 3 and Figure 4) ANOVA with Tukey post hoc tests. The statistics of qPCR data (Figure 3 and Figure 4) was done on log2-transformed data. Significance was accepted at *p* < 0.05. The LC50 for Rg1 on Figure S1 was performed in the R software environment for statistical computing and graphics, using the ‘drc’ package [43].

## 3. Results

### 3.1. Ginsenoside Rg1 Has Anti-Inflammatory Effects that Are Mediated by the Gr

To investigate the anti-inflammatory effect of Rg1 (chemical structure shown in Figure 1A), we studied its effect on the migration of leukocytes in the zebrafish tail fin amputation model at 4 h post amputation (hpa) (Figure 1B). We tested Rg1 at a concentration range from 30 to 180 μM (2 h treatment before amputation, 4 h treatment after amputation), and we used the synthetic GC beclomethasone as a reference at a concentration of 25 μM (previously shown to inhibit neutrophil migration [32]). We found that Rg1 inhibited neutrophil migration to the amputation site to a similar extent as beclomethasone when used at concentrations of 120 μM or higher (Figure 1C). To test the toxicity of Rg1, a Fish Embryo Acute Toxicity Test (FET) was performed in which zebrafish embryos were exposed to a concentration range of Rg1 until 96 hpf, and the effects on hatching and survival were determined (Figure S1). An LC50 of 155.0 ± 1.7 μM was determined. No effects were observed at 100 μM, and minimal effects at 120 μM. Based on this result, and because we use relatively short (6 h) treatments, we selected 120 μM as the test concentration for experiments in this study (this treatment showed smaller effects in the FET than 25 μM beclomethasone, which we routinely use in this type of experiment [32,36]). Given the relatively low affinity of Rg1 for the human GR [24], it is not surprising that high doses are required for Gr-mediated effects in our studies. Next, we compared the anti-inflammatory effects of Rg1 (120 μM) and beclomethasone (25 μM) on both neutrophils and macrophages. In control larvae treated with vehicle, an average of 14.3 ± 0.8 neutrophils and 15.7 ± 1.1 macrophages accumulated in the wounded area, and beclomethasone showed a significant suppression of the neutrophil migration at 4 hpa (9.6 ± 1.0), but did not suppress the migration of macrophages (15.0 ± 1.0) as previously shown [32,36] (Figure 1D–F). A similarly specific inhibitory effect on the migration of neutrophils was observed for the Rg1 treatment, resulting in a significant suppression of the neutrophil migration at 4 hpa (7.1 ± 0.6) and no effect on the migration of macrophages (11.0 ± 0.9, Figure 1D–F). In addition to its effect on migration, we examined whether Rg1 affected the total number of neutrophils and macrophages in the larvae. For this purpose, cell numbers were determined in the whole tail fin area (posterior to the yolk extension, Figure 1B), and the results of this measurement showed no effect of Rg1 on the total number of macrophages and neutrophils (Figure 1G,H). From these experiments, we conclude that Rg1, similarly to beclomethasone, inhibits the migration of neutrophils, but leaves the migration of macrophages unaffected.

Previously, we have shown that GCs inhibit neutrophil migration upon tail fin amputation via activating the Gr [32]. Since it has previously been reported that Rg1 is a functional GR ligand [23,24,25], we studied whether the observed effects of Rg1 are dependent on the presence of the Gr. For this purpose, we used the *gr^s357^* mutant zebrafish line (hereafter referred to as *gr^−^*), which has a point mutation in the gene encoding the Gr which makes the receptor transcriptionally inactive [40]. Both beclomethasone and Rg1 significantly inhibited neutrophil migration at 4 hpa in *gr^+/+^* larvae, while neither of them inhibited neutrophil migration in *gr^−/−^* larvae (Figure 2). These results demonstrate that the effects of both Rg1 and beclomethasone on neutrophil migration are mediated by the Gr.

### 3.2. Rg1 And Beclomethasone Differentially Regulate Gene Expression

To further investigate the effects of Rg1 in the tail fin amputation assay, we determined the mRNA levels of 17 immune-related genes by qPCR at 4 hpa, in vehicle-, beclomethasone-, and Rg1-treated larvae. Three of these genes encoded receptors and signaling intermediates upstream in the inflammatory pathway: *tlr2*, *tlr4ba*, *nfkbiaa*. The results showed that beclomethasone treatment increased the expression of all 3 genes at 4 hpa, but that Rg1 did not significantly alter the amputation-induced expression of either of these genes at 4 hpa (Figure 3A). Three of the studied genes encoded proinflammatory cytokines: *il1b*, *il6*, and *tnfa*. Neither Rg1 nor beclomethasone regulated the expression of *tnfa* at 4 hpa, and both Rg1 and beclomethasone inhibited the expression of *il1b* and *il6* (Figure 3B). Two of the investigated genes encoded proteins with a role in inflammation and tissue regeneration: *mmp9*, *mmp13a*. Our results show that beclomethasone treatment downregulated both *mmp9* and *mmp13* expression at 4 hpa, whereas Rg1 treatment attenuated the amputation-induced expression of *mmp9*, but did not affect the amputation-induced expression of *mmp13* at 4 hpa (Figure 3C). Nine of the investigated genes encoded chemokines and chemokine receptors: *cxcl8a*/*il8* with receptors *cxcr1* and *cxcr2*, *cxcl18b* and its receptor *cxcr2*, *cxcl11aa* and its receptor *cxcr3.2*, receptor *cxcr4b*, *ccl2* and its receptor *ccr2* (Figure 3D,E). The expression of *cxcl11aa*, *ccl2*, and *cxcl18b* was decreased by both Rg1 and beclomethasone at 4 hpa, whereas the expression of *cxcl8a*/*il8* was decreased by beclomethasone, but not Rg1 (Figure 3D). Beclomethasone upregulated the expression of *cxcr1* and *cxcr2* (Figure 3E), and the increase was not observed after Rg1 treatment (Figure 3E). No effects of either beclomethasone or Rg1 were observed on the amputation-induced expression of *ccr2* and *cxcr3.2*, whereas the expression of *cxcr4b* was exclusively downregulated by Rg1 (Figure 3E).

These data show that for many genes, beclomethasone and Rg1 have the same transcriptional effects. For five genes, their upregulation by amputation was suppressed by both beclomethasone and Rg1 (*il1b*, *il6*, *mmp9*, *cxcl18b*, *cxcl11aa*), and four genes were not regulated by either beclomethasone or Rg1 (*tnfa*, *ccl2*, *ccr2*, and *cxcr3.2*). Interestingly, seven genes showed regulation by beclomethasone and not by Rg1. Five of these genes (*tlr2*, *tlr4b*, *nfkbiaa*, *cxcr1*, and *cxcr2*) showed upregulation by beclomethasone, and the remaining two (*mmp13* and *cxcl8/il8*) were downregulated. Only one gene (*cxcr4b*) showed (down)regulation by Rg1 and not by beclomethasone.

Since most of the differences in gene regulation between beclomethasone and Rg1 involved genes that were upregulated by beclomethasone and not affected upon Rg1 treatment, it could be suggested that Rg1 activates the Gr in such a way that it is able to transrepress gene transcription, but is not able to transactivate. To further study the transcriptional regulation by Gr upon Rg1 treatment, the mRNA levels of two Gr target genes, known to be activated by transactivation, were studied: *pck1* and *fkpb5* (Figure 4A,B, respectively). Beclomethasone induced a significant upregulation of both genes, but Rg1 did not affect the transcription rate of these genes, in line with the hypothesis that the Rg1-activated Gr does not have transactivational activity.

### 3.3. Rg1 Does Not Inhibit Tissue Regeneration, Unlike Beclomethasone

**Figure 5:**
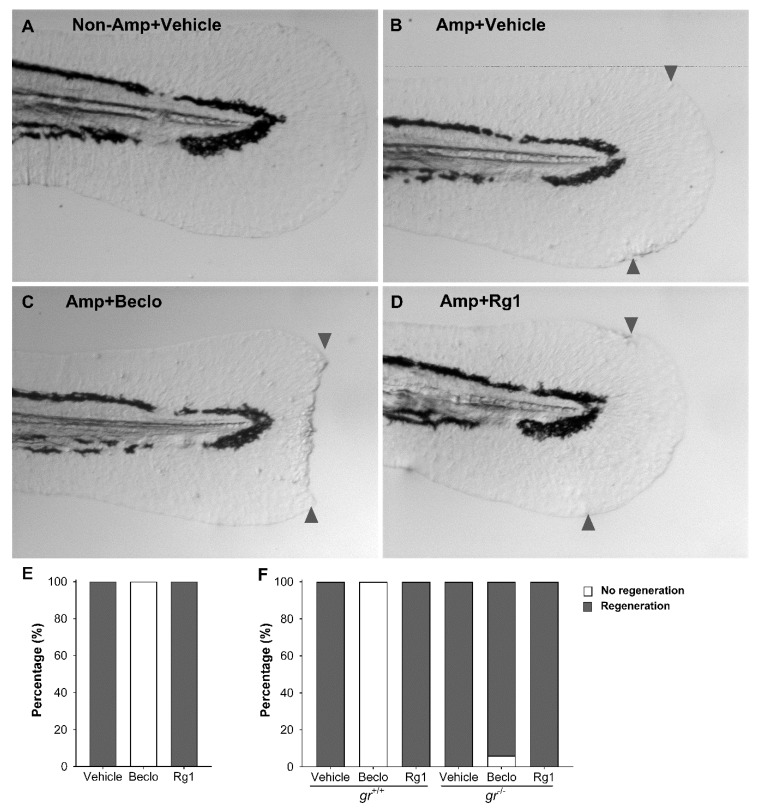
Effects of beclomethasone and Rg1 on the regeneration of the tail fin. (**A**–**D**) Representative images of the tail fins of zebrafish larvae at three days after amputation (Amp) or without amputation (Non-Amp) and treated with vehicle, beclomethasone (Beclo), or Rg1. The approximate location of the amputation site is indicated with arrowheads on the dorsal and ventral side of the tail fin. The images show full regeneration of the tail fin in the vehicle and Rg1 treated larvae, and a total lack of regeneration in the Beclo group. (**E**) Quantification of the tail fin regeneration in zebrafish larvae treated with Vehicle, Beclo, or Rg1 (*n* = 90 larvae per group, accumulated from three independent experiments. (**F**) Quantification of the tail fin regeneration in Gr mutant (*gr^−/−^*) and control (*gr*^+/+^) larvae treated with vehicle, Beclo, or Rg1 (*n* = 90 larvae per group, accumulated from three independent experiments).

To study the effects of Rg1 on tissue regeneration, we performed tail fin amputation at 2 dpf, and treated 2 h before and 4 h after the amputation with vehicle, beclomethasone, or Rg1, and assessed progression of tail fin regeneration progression at 5 dpf compared to the nonamputated group treated with vehicle. We found that 100% of larvae treated with vehicle were able to regenerate their amputated tail fins (Figure 5B,E). Rg1 treatment did not affect the ability to regenerate the tail fins (Figure 5C,E). In contrast, the larvae treated with beclomethasone did not regenerate their amputated tail fins, as previously shown [20] (Figure 5D,E).

To determine if regeneration of the amputated tail fins was dependent on the Gr, we studied regeneration in Gr mutant larvae. We found that amputated *gr^+/+^* and *gr^−/−^* larvae treated with Rg1 or vehicle were able to regenerate their amputated tail fins completely (Figure 5F). Additionally, we found that *gr^+/+^* larvae treated with beclomethasone lost their ability to regenerate their amputated tail fins, while 94% of *gr^−/−^* larvae treated with beclomethasone regenerated their amputated tail fins (Figure 5F). These results demonstrate that the inhibition of regeneration by beclomethasone is mediated by the Gr.

Taken together, our results demonstrate that Rg1 has a potent anti-inflammatory activity which is dependent on the Gr in the tail fin amputation assay, while Rg1 has no adverse effects on tissue regeneration, despite that it was used at higher concentration than beclomethasone in our study.

## 4. Discussion

GCs are frequently used for treating an overactive immune system, but their clinical use is limited by a wide range of side effects, including wound healing disorders and tissue degeneration. Therefore, there is an urgent need for the development of new, more selective GCs with similar anti-inflammatory effects, but with strongly reduced or no adverse effects. Pharmacological research on ginsenosides has presented Rg1, the most abundant ginsenoside in *Panax ginseng*, as a GR ligand with anti-inflammatory activity [23,24,25]. In the present study, we have explored the potential of Rg1 as a selective Gr agonist in zebrafish larvae. Our data demonstrate that Rg1 has potent anti-inflammatory effects similar to beclomethasone. Both Rg1 and beclomethasone inhibited wound-induced migration of neutrophils, in a Gr-dependent manner. However, analyses of gene expression showed that Rg1 has different effects from beclomethasone on the expression of several immune-related genes. Importantly, Rg1 does not inhibit tissue regeneration, whereas beclomethasone inhibits the regeneration of the amputated tail fins. These results demonstrate a selective mode of action of Rg1, whereby inflammation is dampened without side effects on tissue healing and regeneration.

The specific inhibitory effect of Rg1 on neutrophil migration is in line with previous reports, which showed that Rg1 is a ligand for the human GR [24], and that Gr activation in our zebrafish tail fin amputation specifically suppresses the migration of neutrophils and leaves the macrophage migration unaffected [32]. Rg1 has been shown to bind to the human GR with an affinity 10–100 fold lower than dexamethasone, thereby inducing transcriptional and nontranscriptional actions of the receptor in cultured cells [23,25]. We have demonstrated that the zebrafish Gr is well conserved between humans and zebrafish, and that the zebrafish Gr is structurally and functionally very similar to its human orthologue [28]. It has been shown that various GCs, such as dexamethasone, beclomethasone, prednisolone, and hydrocortisone, display a suppressive effect on the migration of neutrophils (and not macrophages) in the zebrafish tail fin amputation assay through activation of the Gr [36]. In accordance with these findings, in the present study, we have demonstrated that the inhibition of neutrophil migration upon Rg1 treatment is mediated by the Gr. Since Rg1 is the most abundant ginsenoside in Ginseng Radix, the activity of Rg1 may underlie the anti-inflammatory effects of Ginseng preparations [26,45].

Rg1 has previously been shown to activate the GR and elicit both transcriptional and nontranscriptional effects like other GCs [23,24,25,26,46]; however, in the present study, we show that Rg1 activation of Gr elicits different effects from a classical GR agonist like beclomethasone. Rg1 treatment did not interfere with regeneration of the zebrafish tail fin after wounding, whereas beclomethasone treatment completely inhibited regeneration. A similar type of selective activity of Rg1 in vivo was previously found in mice [26]. Rg1 was shown to have anti-inflammatory effects in mouse models for (chronic) collagen-induced arthritis and (acute) zymosan-induced paw inflammation, without adverse effects on glucose levels or osteoblast proliferation and differentiation [26].

Rg1 was not only shown to lack the side effect of beclomethasone on tissue regeneration in our study; it also appeared to modulate gene regulation more selectively than beclomethasone. In a previous study, we have shown that beclomethasone attenuates almost the entire transcriptional response to amputation [32]. In the present study, both beclomethasone and Rg1 were shown to inhibit the amputation-induced increases in the expression of the genes encoding Il1b, Il6, Cxcl18b, Cxcl11aa, and Mmp9. However, the (modest) suppression of the genes encoding Cxcl8/Il8 and Mmp13 upon beclomethasone treatment was not observed after Rg1 treatment. Moreover, the increased expression of the genes for Tlr2 and Tlr4-a, NF-κB inhibitor α-like protein A, Cxcr1, and Cxcr2 was exclusively seen after beclomethasone treatment.

The mechanism underlying this selective action is still unclear. This different action may be a result of a different conformation of the Gr ligand-binding domain upon Rg1 binding. This conformation may preferentially induce transrepression, rather than transactivation activity. Previously, such transrepression-selective GR activation has been shown for different ginsenosides (20S)-Protopanaxadiol (PPD) and (20S)-Protopanaxatriol (PPT) [47], but available data for Rg1 are conflicting [24,26,46]. Our results on gene transcription generally support this type of selectivity for Rg1, since all studied genes of which the expression was upregulated by beclomethasone did not show this upregulation in the presence of Rg1, whereas the vast majority of genes downregulated by beclomethasone were also downregulated upon treatment with Rg1. When we studied the expression of *pck1* and *fkbp5*, Gr target genes which are known to be activated by transactivation, we demonstrated that, in contrast to beclomethasone, Rg1 did not increase the transcription of these genes, confirming the lack of Gr transactivation activity upon Rg1 treatment. Since transactivation is the mechanism generally linked to the side effects of GC therapy, a lot of research is currently focused on the development of novel GR agonists that selectively induce the transrepression activity of the receptor [48]. Studies on the selectivity of Rg1 and other ginsenosides such as PPD and PPT and the underlying molecular mechanisms, may open up new avenues in this research on novel selective GR agonists.

Moreover, the data presented here provide some insights into the molecular mechanisms underlying neutrophil migration and tissue regeneration. First, our results suggest that neutrophils are not a crucial factor in tissue regeneration, as after treatment with Rg1, neutrophil migration was inhibited, but the larvae were still able to regenerate their amputated tail fins. This conclusion is supported by a previous study using the zebrafish tail fin amputation assay in which ablation of the neutrophils did not affect regeneration of the fin, whereas ablation of macrophages did [49]. Second, we show that beclomethasone treatment for only 4 h after the amputation is sufficient to block regeneration, which indicates that the early response to wounding plays a crucial role in the regenerative process in this assay, in line with previous observations [50]. Third, the beclomethasone-induced decrease in *cxcl8*/*il8* expression is not required for the inhibition of neutrophil migration, whereas the observed attenuation of *cxcl18b* expression (one of the most highly induced proinflammatory genes upon amputation) could be an important mechanism underlying this inhibition [32]. Fourth, the lack of downregulation of *cxcl8*/*il-8* and *mmp13* by Rg1 may play a role in its inability to block tissue regeneration, since Cxcl8/Il-8 and Mmp13 have been reported to play a role in tissue remodeling and regeneration by enhancing processes like keratinocyte migration, angiogenesis, and wound healing [51,52].

## 5. Conclusions

Here, we present evidence that the ginsenoside Rg1 acts as a selective GR agonist in our zebrafish model, with potent anti-inflammatory effects but without affecting tissue regeneration, opening up a novel road towards the development of improved anti-inflammatory GCs. Several other ginsenosides, structurally similar to Rg1, have been identified that can also be explored for application as selective GR agonists. Furthermore, in future work, the zebrafish may serve as a translational model to unravel the mechanisms underlying the selectivity of Rg1 and related ginsenosides, and for screening of compounds to evaluate their potential for clinical use.

## Supplementary Materials

The following are available online at https://www.mdpi.com/2073-4409/9/5/1107/s1, Table S1: Sequence of primers for the qPCR analysis. Figure S1: Fish Embryo Acute Toxicity Test (FET) for Rg1.
