# Supplementary material for: Ginsenoside Rg1 Acts as a Selective Glucocorticoid Receptor Agonist with Anti-Inflammatory Action without Affecting Tissue Regeneration in Zebrafish Larvae
Source: Cells. 2020 Apr 29;9(5):1107. doi: 10.3390/cells9051107 (PMC7290513; doi:10.3390/cells9051107)
Supplement: Supplementary file 1 [file cells-09-01107-s001.pdf]

Table S1. Sequence of primers for the qPCR analysis

	Forward primer sequence	Reverse primer sequence
**Housekeeping gene**		
ppial	CATCCACAACCTTCCCGAACAC	ACACTGAAACACGGAGGCAAAG
**Neutrophil-specific genes**		
cxcl8/il-8	TGTGTTATTGTTTTCCTGGCATTTC	GCGACAGCGTGGATCTACAG
cxcl18b	TCTTCTGCTGCTGCTTGCGGT	GGTGTCCCTGCGAGCACGAT
cxcr1	CGTTTGTTCCCGACGAGAAG	CAGCGGATGCCATTGTGAT
cxcr2	TGACCTGCTTTTTTCCCTCACT	TGACCGGCGTGGAGGTA
**Macrophage-specific genes**		
ccl2	GTCTGGTGCTCTTCGCTTTC	TGCAGAGAAGATGCGTCGTA
cxcl11aa	ACTCAACATGGTGAAGCCAGTGT	CTTCAGCGTGGCTATGACTTCCAT
ccr2	TGGTCGTCTGGGTCCTCATT	ACCTGCCCAAAATCCACTCA
cxcr3.2	CCTCTGTTGGTAATGCTGTATTGC	ACACGATGACTAAGGAGATGATG
cxcr4b	GCGACCTCTCAGTCAGCAAT	TCACAAGCACCACAAGTCCA
**Inflammation-related genes**		
tnfα	ACCAGGCCTTTTCTTCAGGT	TTTGCCTCCGTAGGATTCAG
nfkbiaa	CTTGGGCTAAAGTAGTCACCG	GATGGCAAGGTGCAGATACGTG
tlr2	CAGGATGAGACTCGTAGGAACA	CCATTCAACAAGTAGGTAGCACT
tlr4ba	CCTATTTTTGCAGAGCCCTTCACA	GTGTGTAACTCTACCAAATGACA
il-1b	CATAAACACCTTCGAGTCCG	TCTTTCCTGTCCATCTCCACCA
il-6	AGACCGCTGCCTGTCTAAA	TTTGATGTCGTTCACCAGGA
**Matrix metalloproteinase-related genes**		
mmp9	CATTAAAGATGCCCTGATGTATCC	AGTGGTGGTCCGTGGTTGAG
mmp13a	ATGGTGCAAGGCTATCCCAAGAGT	GCCTGTTGTTGGAGCCAAACTCAA
***Gr* target genes**		
fkbp5	TCTGCCAGCACAAGATTCGTGAGC	GACCCTGCTTATTCTGATCGGAAA
pck1	CAGGGCGATCTGGCGTCTCT	CTGCTGTCGATGAACTCCCG

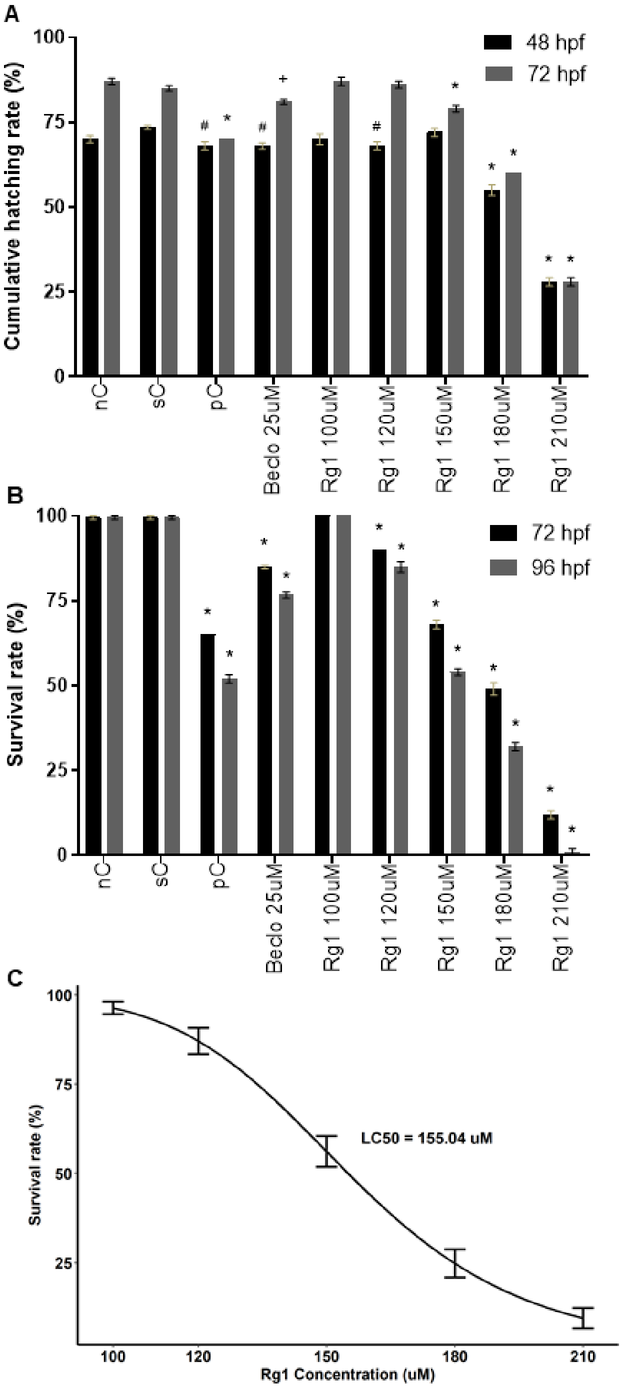

Figure S1. Fish Embryo Acute Toxicity Test (FET) for Rg1. Embryos were exposed to different concentrations of Rg1 to test the toxicity of this compound. A negative control (nC), solvent control (sC), and a positive control (pC) group were included in the experiment, as well as a group exposed to 25 μM beclomethasone. A) Hatching rate (%) at 48 and 72 hpf. B) Survival rate (%) at 72 and 96 hpf. Statistical significance ($p<0.05$) in A and B is indicated by: * (different from nC and sC), + (different from nC), and # (different from sC). C) Survival rates plotted against the Rg1 concentration. Upon curve fitting, the LC50 was determined. Data shown are means ± SEM.
